# Prevalence and Morphology of the Palatal Bone Reservoir in the Posterior Maxilla as an Alternative to Maxillary Sinus Lift: A Cross-Sectional Retrospective Analysis Determined by Helical CT Scan

**DOI:** 10.3390/dj14050260

**Published:** 2026-04-30

**Authors:** Andrei Krasovsky, Ahmad Hija, Husam El Khatib, Ori Blanc, Amir Bilder, Chaim Ohayon, Tal Capucha, Omri Emodi

**Affiliations:** 1 Oral and Maxillofacial Surgery, Rambam Medical Care Campus, HaAliya HaShniya St. 8, Haifa 3109601, Israel; 2 Ruth & Bruce Rappaport Faculty of Medicine, Technion-Israel Institute of Technology, Haifa 3200003, Israel; 3 Department of Oral Pathology, Oral Medicine and Maxillofacial Imaging, School of Dental Medicine, Faculty of Health and Medical Sciences, Tel Aviv University, Tel Aviv 6997801, Israel; dr.husamkhatib@gmail.com

**Keywords:** palatal bone reservoir, tilted implants, virtual implant planning, bone density, Hounsfield units

## Abstract

**Background:** Maxillary sinus lift is among the most common preprosthetic procedures in the posterior maxilla due to alveolar ridge resorption and the maxillary sinus pneumatization. It often extends treatment duration, significantly increases costs, and is not without complications. **Objective:** To explore the prevalence and morphology of the palatal bone reservoir as a viable site for dental implant insertion, offering a conservative alternative to avoid maxillary sinus lift. **Methods:** DICOM data sets from helical CT of maxillofacial trauma patients aged 50 years and older were used to perform virtual dental implant positioning in the edentulous second premolar, first molar, or second molar areas using ImplaStation software (version 5.3.2; ProDigiDent, Inc., Scottsdale, AZ, USA). A 3D Slicer software (version 5.3.2; ProDigiDent, Inc., Scottsdale, AZ, USA) was used to calculate the volume of the palatal bone reservoir and identify its mean density. The density of the residual alveolar process was also identified and compared with that of the previous one. **Results:** A total of 1822 maxillofacial trauma cases with helical CT between 2015 and 2025 were retrieved. After exclusion, 305 cases were analyzed. A total of 65 implants were virtually positioned in 50 patients. The mean volume of the palatal bone reservoir was 229 ± 139.2 mm^3^ with a mean radiodensity of 546.7 ± 159.6 HU. The mean radiodensity of the residual alveolar process was 286.3 ± 118.0 HU. The palatal bone reservoir was significantly denser than the residual alveolar process (95% CI [184.2, 336.6]; *p* < 0.01). **Conclusions:** The presence of a palatal bone reservoir is not uncommon and can offer a more conservative alternative for implant placement, potentially increasing primary stability and facilitating immediate loading.

## 1. Introduction

Global prevalence and incidence of tooth loss begin to increase gradually in the middle-aged population, mainly due to untreated caries and periodontal disease [1]. Available solutions range from complete/partial removable dentures, fixed partial dentures, and dental implants.

In the posterior maxillary area, maxillary sinus pneumatization often necessitates preprosthetic treatment, such as crestal and lateral window sinus lift procedures. Sinus augmentation is a very common surgical procedure in the posterior maxilla, involved in over half of maxillary posterior implant cases today [2]. The typical age for a sinus lift procedure is approximately 50 years [3,4]. Numerous classifications of sinus lifts have been published in the literature, primarily focusing on residual bone height and sinus contours resulting from alveolar bone resorption and sinus pneumatization [5,6,7]. The primary objective of these classifications is to provide treatment recommendations regarding the need for a sinus lift procedure and the specific techniques to be used.

Although sinus lift is generally considered a safe and predictable procedure, it is not without complications [8]. Crestal sinus lift is a faster, less invasive, but technically demanding procedure. The most common documented complication is perforation of the Schneiderian membrane during malleting with an osteotome [9]. Benign paroxysmal positional vertigo and implant displacement into the maxillary sinus are also documented [9]. Lateral window sinus lift complications include intraoperative Schneiderian membrane perforation and uncontrolled bleeding, and postoperative wound infection, sinusitis, graft exposure, graft infection, and flap dehiscence [10].

Sinus lift may substantially increase the procedure’s cost and prolong the overall treatment period, especially when done in a two-stage approach. Several surgical alternatives for sinus lift have been documented in the literature and used in clinical practice. Short, tilted, and pterygoid implants are among the options for graftless procedures [11]. Zygomatic implants can also provide a graftless solution, usually in bilateral cases of severely atrophic maxilla, but in a more complex surgical setting, and with a higher cost [12]. The prosthetic option of using distal cantilevers is another alternative to avoid additional implant placement [13].

The recent literature has suggested palatally positioned dental implants as a viable alternative to maxillary sinus lift [14,15]. However, a comprehensive morphological analysis of the specific bone volume and quality in this region remains lacking. In this study, we explored this less-invasive surgical alternative for posterior maxillary implantation in specific cases of alveolar height deficiency and provided an in-depth morphological assessment of the available bone volume.

## 2. Materials and Methods

### Study Design and Patient Selection

**Figure 1:**
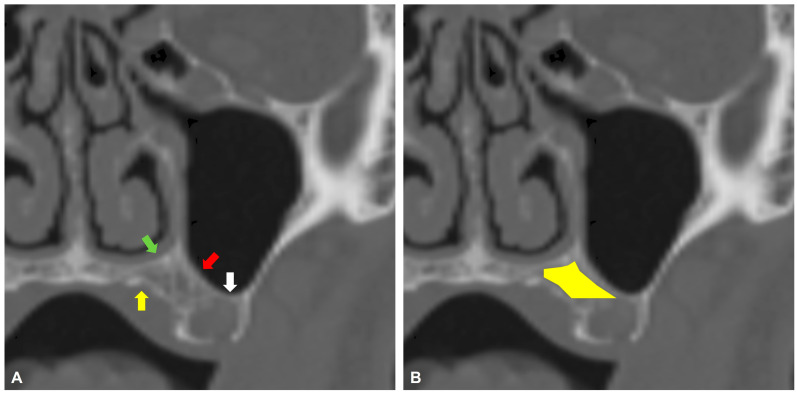
Helical CT coronal view demonstrating the anatomical borders of the palatal bone reservoir. (**A**) The most inferior point of the maxillary sinus floor (white arrow), the medial part of the maxillary sinus floor (red arrow), the horizontal palate (yellow arrow), and the nasal floor (green arrow). (**B**) The area of the palatal bone reservoir is depicted by a yellow polygon.

This retrospective, single-center study included patients with maxillofacial trauma who underwent helical CT scans of the facial area between 2015 and 2025. Inclusion criteria were age ≥ 50 years; a field of view (FOV) for helical CT that included the whole maxillary bone with teeth up to the orbital floor, and at least one missing maxillary tooth, which was either the second premolar, first molar, or second molar. Included patients were screened for the presence of trabecular bone stock volume located palatally to the medial part of the maxillary sinus floor, superiorly to the maxillary sinus floor’s lowest point, and buccally to the horizontal palate, as visualized on coronal helical CT view (Figure 1). Only helical CTs with a 1 mm slice thickness were included. Exclusion criteria were patients with fractures involving the maxillary bone—in unilateral cases, only the affected side was excluded, in bilateral cases, the patient was excluded. Also, patients with an alveolar crest height of ≥8 mm inferior to the maxillary sinus were excluded. Helical CTs with contrast were excluded from further analysis.

## 3. Virtual Implants Positioning

**Figure 2:**
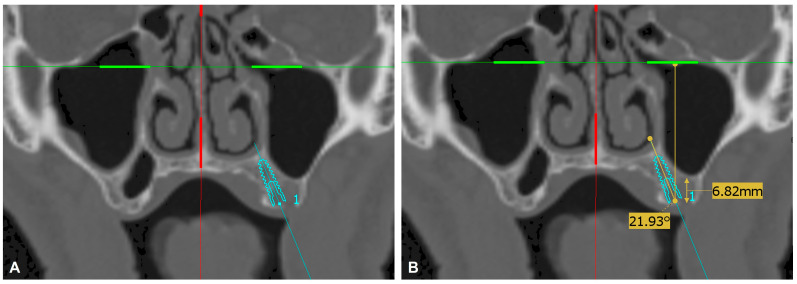
Helical CT coronal view. (**A**) An ICE implant 3.75/13 mm is virtually placed at the position of the first left maxillary molar, not penetrating the cortical borders. (**B**) The long axis angle of the implant is less than 30°, and the height of the alveolar crest is less than 8 mm.

To functionally evaluate the palatal bone reservoir at the posterior maxilla, in the areas of missing second premolars and first and second molars, virtual dental implant positioning was performed using ImplaStation software (version 5.3.2; ProDigiDent, Inc., Scottsdale, AZ, USA) on anonymized DICOM files from maxillofacial trauma patients. Each virtual implant position was evaluated and approved by the same attending surgeon. An ICE implant (Alpha-Bio Tec, Modi’in-Maccabim-Re’ut, Israel.) with a diameter of 3.75 mm and variable lengths of 10, 11.5, and 13 mm was used in all cases as a benchmark. The implant was positioned according to the following criteria: implant long axis angle < 30° relative to the axis perpendicular to the infraorbital line; implant fully confined in trabecular bone, not penetrating the cortical borders (Figure 2). The implant virtual anatomical position was defined by the analogous missing tooth (second premolar, first molar, or second molar) when one tooth was missing. In cases of multiple missing teeth and difficulty in defining the implant position solely by its proximity to the remaining teeth, the anatomical location of the first maxillary molar was localized in the alveolar crest area just below the infrazygomatic crest [16] that being a reference area for localizing the second premolar and second molar teeth. If the implant virtual positioning met the above criteria, the case was then subjected to the radiographic morphological analysis. Otherwise, the case was considered inadequate for the placement of a palatal implant.

## 4. Radiographic Analysis

The 3D Slicer software (version 5.8.1; available at http://www.slicer.org, accessed on 10 November 2025) was used to evaluate the palatal bone reservoir morphology in cases where virtual implant positioning was possible. First, patients’ DICOM files were imported into the software and reviewed in multiplanar views. Two different regions of interest (ROI) were evaluated: The ROI1 for palatal bone reservoir assessment was defined anatomically using two reference boundaries: (1) above a horizontal plane aligned with the base of the maxillary sinus, and (2) medial to a straight line tangential to the palatal aspect of the alveolar bone. The ROI2 for the residual alveolar process was defined below a horizontal plane aligned with the base of the maxillary sinus. Within these ROIs, segmentation was performed to isolate trabecular (spongious) bone, with the surrounding cortical shell deliberately excluded to avoid contamination by cortical bone. The segmentation was refined iteratively in axial, coronal, and sagittal planes to ensure continuity of the trabecular compartment and adherence to the predefined anatomical limits. Following segmentation, quantitative outputs were generated directly within 3D Slicer. Bone volume of ROI1 was calculated from the final segmented labelmap using the image spacing metadata, and attenuation metrics were extracted in Hounsfield units (HU) from the segmented voxels for ROI1 and ROI2. For each segmented ROI, the software reported HU summary statistics (minimum, maximum, mean, and standard deviation) alongside the corresponding volume. Finally, a paired *t*-test was conducted to assess the difference in HU between ROI1 and ROI2.

## 5. Statistical Analysis

IBM SPSS Statistics version 27.0 (IBM Corp., Armonk, NY, USA) was used for the statistical analysis. Also, quantitative analysis of the bone densities was performed in 3D Slicer, as described previously.

## 6. Results

A total of 1822 maxillofacial trauma cases with helical CT scans were identified at our health care campus between 2015 and 2025. Trauma cases included mandibular symphyseal and parasymphyseal, body, angle, coronoid, subcondylar, and condylar fractures; Le Fort 1, 2, and 3 fractures; zygomatic complex and zygomatic arch fractures; orbital fractures; nasal and naso-orbito-ethmoid fractures; and combinations of the above. Of these, 398 patients were ≥ 50 years old. After removing duplicate records for returning patients and follow-up CTs, and screening for 1 mm slice-thickness helical CTs with appropriate FOV, a total of 305 cases remained. These cases were evaluated for the presence of a palatal bone reservoir at the posterior maxilla. Fifty patients (16.4%) had sufficient posterior palatal bone volume for virtual positioning of the ICE implant (Alpha Bio Tec.) using the previously described method. Demographic data of 305 patients are presented in Table 1. The distribution of virtual implant placement at the palatal bone reservoir in the posterior maxilla is presented in Table 2. A total of 65 implants were virtually positioned in 50 patients. In 15 patients, one implant was positioned bilaterally. In 2 (4%) patients with sufficient posterior palatal bone volume on one side, the contralateral side had a fracture line in the posterior maxilla. It was therefore not included in the morphologic analysis. In 8 (3%) patients with insufficient posterior palatal bone volume on one side, the contralateral side had a fracture line in the posterior maxilla. It was therefore not included in the morphologic analysis.

**Figure 3:**
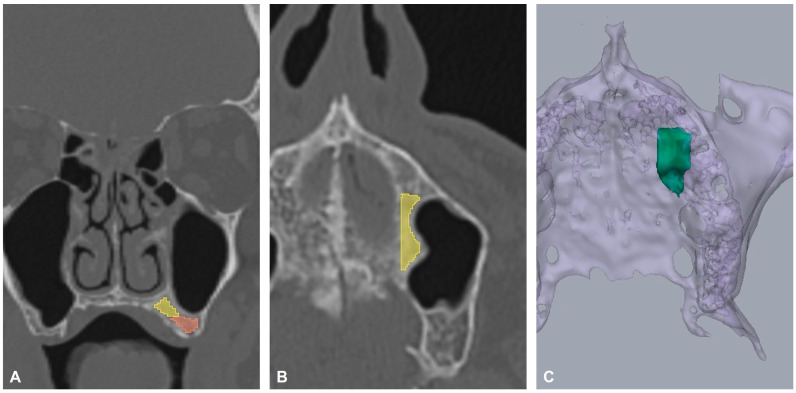
Representation of the segmented palatal bone reservoir (ROI1) and residual alveolar process (ROI2). (**A**) The yellow color represents ROI1 and the red color represents ROI2 on the coronal view. (**B**) ROI1 is represented in yellow on the axial view. (**C**) The green color represents the total bone volume in the 3D view.

Radiographic volumetric and morphologic analysis of the segmented palatal bone reservoir is summarized in Table 3. The mean volume of the bone stoke was 229 ± 139.2 mm^3^. Figure 3 provides the 2D and 3D representations of the segmented palatal bone reservoir and a 2D representation of the residual alveolar process. The mean radiodensity in ROI1 (546.7 ± 159.6 HU) was significantly higher than in ROI2 (286.3 ± 118.0 HU), with a mean difference of 260.4 HU (95% CI [184.2, 336.6]; *p* < 0.01).

## 7. Discussion

This study analyzed the prevalence and morphological characteristics of the volume and density of the anatomical palatal bone reservoir variant. Furthermore, virtual positioning of dental implants within this bone volume was tested. The presence of a palatal bone reservoir can enable a more conservative, rapid method for inserting dental implants in a palatally inclined position, thus avoiding additional preprosthetic surgery. Anteriorly and posteriorly tilted implants are well known as an effective and safe alternative for the sinus lift procedure [17]. However, this strategy is only feasible in cases of multiple missing teeth, as such an implant inclination is only possible with extra edentulous anteroposterior space. Otherwise, it would damage the adjacent tooth roots. In cases of a single missing posterior tooth, only the palatal implant inclination option would be feasible to ensure the safety of adjacent teeth. In our analysis, we limited virtual implant insertion into the palatal bone reservoir to a palatal angle below 30°, as an angle of 30° and above in single implants significantly increases the risk of bone loss [18]. The ICE implant (Alpha Bio Tec.) was selected for virtual planning because of its moderately tapered body and core, which better accommodate the narrow zone between the palate and the maxillary sinus floor. In the literature, the upper threshold for the classification of short implants is established at less than 10 mm [11]. In this study, virtual short dental implants were excluded from the analysis; therefore, the minimum implant length was 10 mm. Virtual implants with a narrow platform were also excluded from the analysis, and only implants with a 3.75 mm diameter were used. It is worth noting that ImplaStation software (version 5.3.2; ProDigiDent, Inc., Scottsdale, AZ, USA) currently offers a free virtual implant planning feature. Clinicians can easily use this modality to identify the palatal bone reservoir and virtually plan the implant diameter, length, and angulation. Freehand dental implant insertion in a palatal angulation may be challenging, as it increases the risk of maxillary sinus perforation or compromising the available bony envelope. Hence, we strongly recommend a guided surgery protocol in such cases.

We choose to examine the population aged 50 years and older, since in the developed world, periodontal disease becomes an additional significant reason for tooth loss, alongside caries, in the 6th decade of life [19]. We evaluated the palatal bone reservoir at the anatomical positions of the second premolar and the first and second molars. The first premolar was excluded from evaluation because, in most cases (58%), its location coincided with only the anterior border of the maxillary sinus [20].

The study population comprised patients with maxillofacial trauma, as those patients are routinely referred for helical CT in the emergency department. Helical CT allows for the evaluation of radiodensity in HU and provides more reliable values than Cone Beam Computed Tomography (CBCT) [21,22]. Also, the HU unit system can be easily compared with classical bone density measurements. According to Shapurian et al., the mean density in the posterior maxilla is 333 ± 199 HU [23]. Our results for the residual alveolar process (ROI2) were slightly lower (286.3 ± 118.0 HU) [23]. This can be explained by the fact that our density evaluation method did not include the cortical portion of the bone. Interestingly, the mean density of palatal bone reservoir (ROI1) of (546.7 ± 159.6 HU) resembles that of the anterior mandible (559 ± 208 HU) as found by Shapurian et al. This finding can have important clinical implications for choosing to use the palatal bone reservoir to improve primary implant stability and consider immediate loading.

The literature has scarcely addressed the palatal bone volume at the posterior maxillary as a potential additional location for implant insertion [14]. Only one study by Kaya et al. used CBCT to virtually assess the feasibility of implant insertion in this area in patients with only one missing posterior tooth, excluding the first premolar [15]. No bone morphology was examined in this work. Similarly, in our work, only implants with a palatal tilt of less than 30° were considered as valid. Also, in both studies, the highest rate of virtual implants was in the second premolar region, and the lowest rate was at the second molar region. However, their study identified a considerably higher percentage (57.5%) of available palatal bone suitable for implant placement compared to our findings (21.3%). Several methodological differences can explain this. First, Kaya et al. used a narrower implant (Straumann 3.3–8 mm, BLT, Basel, Switzerland). Second, the implant placement was recorded as invalid if it perforated the maxillary sinus or the nasal cavity. We use a stricter methodology and require the implant not to touch the cortical boundaries.

The limitations of this study include a negligible number of patients with fracture lines crossing one side of the posterior maxillary area; these sides are not included in the morphologic analysis. Only implants with a diameter of 3.75 mm were tested, and short implants were excluded from the analysis. Further research, testing a range of implant diameters, is warranted to obtain higher-resolution data. This study presents only the results of virtual implant positioning and digital morphological analysis. Clinical studies of an implant inserted into the palatal bone reservoir will add data regarding primary stability, insertion torque, and the potential for immediate loading. Finally, determining the most appropriate implant shape for engagement with the palatal bone reservoir will also be highly valuable.

## 8. Conclusions

The palatal bone reservoir in the posterior maxilla is not uncommon and can offer a more conservative alternative for implant placement, avoiding preprosthetic procedures such as a maxillary sinus lift. This area is denser than the residual alveolar process; hence, it can be used to increase primary stability and allow immediate loading.

## Figures and Tables

**Table 1:** Demographic characteristics of the study population.

Variable		Total	Without PBR	With PBR
Gender, n (%)				
	Male	205 (67.2)	183 (71.8)	22 (44)
	Female	100 (32.8)	72 (28.2)	28 (56)
	Total	305 (100)	255 (100)	50 (100)
Mean age, y (SD)		65.8 (10.6)	65.0 (10.2)	69.9 (11.6)
Dental status, n (%)	Missing posterior teeth			
	All		65 (25.5)	37 (74.0)
	Part		43 (16.9)	13 (26.0)
	Non		147 (57.6)	

PBR—Palatal Bone Reservoir.

**Table 2:** Distribution of anatomical virtual implant positions.

Virtual Implant Position		Implant Length			Total, n (%)
		10 mm, n (%)	11.5 mm, n (%)	13 mm, n (%)	
Right side					
	2P	7 (10.8)	4 (6.2)	9 (13.8)	20 (30.8)
	1M	1 (1.5)	5 (7.7)	6 (9.2)	12 (18.5)
	2M	2 (3.1)	2 (3.1)	1 (1.5)	5 (7.7)
Left side					
	2P	4 (6.2)	6 (9.2)	6 (9.2)	16 (24.6)
	1M	4 (6.2)	2 (3.1)	3 (4.6)	9 (13.8)
	2M	2 (3.1)	0 (0.0)	1 (1.5)	3 (4.6)

2P—Second maxillary premolar; 1M—First maxillary molar; 2M—Second maxillary molar.

**Table 3:** Radiographic volumetric and morphologic analysis of palatal bone reservoir.

Variable (n = 50)	Mean (SD)	Range (min–max)	95% CI of Mean	CV %
Volume (mm^3^)	229.8 (139.2)	58.6–602.3	191.3–268.4	60.6
HU mean	546.7 (159.6)	197.2–918.3	502.5–591.0	29.2
HU min	122.0 (78.0)	−66.0–487.0	100.0–143.0	63.9
HU max	1399.0 (420.0)	713.0–3071.0	1283.0–1515.0	30.0

SD—Standard deviation; CI—Confidence interval; CV–coefficient of variations; HU–Hounsfield units.

## Data Availability

The original contributions presented in this study are included in the article. Further inquiries can be directed to the corresponding authors.
